# COVID-19 Related Information on Pediatric Dental Care including the Use of Teledentistry: A Narrative Review

**DOI:** 10.3390/children9121942

**Published:** 2022-12-10

**Authors:** Federica Di Spirito, Alessandra Amato, Maria Pia Di Palo, Giuseppe A. Ferraro, Adone Baroni, Rosario Serpico, Maria Contaldo

**Affiliations:** 1 Department of Medicine, Surgery and Dentistry, University of Salerno, 84084 Salerno, Italy; 2 Department of Neuroscience, Reproductive Science and Dentistry, University of Naples Federico II, 80138 Naples, Italy; 3 Multidisciplinary Department of Medical-Surgical and Odontostomatological Specialities, University of Campania “Luigi Vanvitelli”, 80138 Naples, Italy; 4 Department of Mental Health and Physics and Preventive Medicine, Unit of Dermatology, University of Campania “Luigi Vanvitelli”, 80138 Naples, Italy

**Keywords:** pedodontics, pediatric, oral, dental, teledentistry, COVID-19, SARS-CoV-2, caries, prevention

## Abstract

In addition to the direct impact of the SARS-CoV-2 infection, the COVID-19 pandemic reports multiple effects on people’s health and psycho-physical well-being. In the dental field, oral hygiene sessions, regular dental check-ups, and aerosol-generating procedures were commonly postponed, thus resulting in repercussions on oral health also favored by the changing eating and oral hygiene habits during the multiple lockdowns. Furthermore, dental settings and practices were generally perceived as at high risk for SARS-CoV-2 transmission, thus unsafe, and by general and pediatric dentists themselves. Last, the consequences of stress related to deprivation of social life and playful activities should not be underestimated in children, exposing them to the deleterious effects of bad oral habits, with repercussions on the balanced growth and development of the stomatognathic system. The present work intends to analyze the medium-term and long-term impact of COVID-19 on pediatric oral and dental care provision, reviewing pediatric dentistry practice and oral and dental needs of pedodontics patients during the first peak and the various waves of the COVID-19 pandemic, and lessons learned.

## 1. Introduction

During the first peak of the COVID-19 pandemic, routine dental practice was suspended or postponed, and dental interventions were limited mainly to dental emergencies [1,2] to prevent further spread of SARS-CoV-2 infection and to minimize the risk of developing cross-infections in the dental setting [3,4,5,6].

Emergency and non-emergency oral and dental needs and associated procedures, as classified by the American Dental Association [5,7], have been differentiated by triage of adult and pediatric patients into those that required priority and those that could be deferred to times when the pandemic situation was considered more favorable [8].

According to the American Dental Association (ADA) guidelines [5], emergency dental procedures could only be performed in dental facilities in patients without clinical signs or symptoms of SARS-CoV-2 infection and using appropriate protocols; conversely, patients with obvious symptoms had to be referred to appropriate facilities [4]. Patients who have recovered from COVID-19 could access dental settings after 72 h [6].

As with the adult dental population, pediatric dental patients have suffered the effects of diagnostic and therapeutic delay for oral and dental diseases, favored by a lack of routine checkups and changes in dental hygiene and dietary habits. The present narrative review, therefore, aimed to shed light on these changes and the medium- and long-term impact of COVID-19 on children’s oral health by reviewing the literature on pediatric dentistry and the oral and dental needs of pediatric dental patients during the various and subsequent waves of the COVID-19 pandemic.

## 2. Materials and Methods

The authors searched PubMed, Scopus, Web of Science, and Google Scholar databases through 26 October 2022, to look for scientific articles describing the measures adopted for oral and dental care in children since the initial peak of the COVID-19 outbreak and thereafter and the past and current scenario in pediatric dentistry from epidemiologic and operational perspectives.

A further search addressed tools and applications of teledentistry and Mobile-health care in pedodontics. Those developed during the same period were considered. Their purpose was to allow contact with young patients and their families at a distance, reducing the number of in-person appointments but ensuring the continuity of pediatric dental and oral care. The following keywords were used: Pedodontics OR Pediatrics OR Oral Medicine OR Dentistry OR Teleservice OR M-Health OR Caries OR Prevention AND COVID-19; SARS-CoV-2.

References were exported and managed using Mendeley Reference Manager software.

## 3. Results

### 3.1. Pediatric Oral and Dental Care and the COVID-19 Pandemic

#### 3.1.1. Changes in Pediatric Oral and Dental Care during the First Peak of the COVID-19 Pandemic

Emergency pedodontic procedures and underlying oral and dental needs recorded during 2020 and compared to 2019 showed disparate trends worldwide [5]. Indeed, registered admissions for emergency dental procedures proportionally increased from 2% in 2019 to 3% in 2020 at the emergency department of the Alfred I DuPont Hospital for Children in Wilmington (North Carolina, USA) [9], and similarly raise to 20.8% in South India, while decreased in 2020 compared to 2019 at Children’s Hospital Vittore Buzzi (Milan, Italy) [10].

Specifically, recognized pediatric emergency oral and dental needs comprised early childhood caries, dental pain, facial swelling, and dental traumas [8]. Table 1 summarizes emergency and urgent oral and dental needs in pediatric patients during the first peak of the COVID-19 pandemic.

Conversely, non-emergency oral and dental care in pediatric subjects drastically decreased dramatically compared to 2019, by a factor of seven [11,12], particularly in April, likely due to the tightened restrictions implemented [12]. Coherently, in April, only 30% of pediatric dentists in Poland performed treatments [12]. Table 2 summarizes oral and dental treatment performed in pediatric patients during the first peak of the COVID-19 pandemic.

#### 3.1.2. Pediatric Oral and Dental Care Provision during the Further Waves of the COVID-19 Pandemic

During the subsequent epidemic waves from COVID-19, also related to rapidly emerging new SARS-CoV-2 variants and concomitant with the reduction of restrictive measures, the number of infections in the pediatric population significantly increased [13,14]. Fortunately, the number of deaths from COVID-19 in children has remained low, as the prevalence of the critical and severe forms of the disease [14,15].

However, discontinued long-term treatments, such as orthodontic and prophylactic ones, as well as uncompleted therapies or newly developed pathological conditions, had to be managed, as well as patients’ and parents’ concerns addressed, thus guaranteeing the continuity, timeliness, and, consequently, the effectiveness of pediatric oral and dental care. Pediatric dentistry practice was thus rationalized based on priorities and ergonomically reorganized [7] to reduce the risk of SARS-CoV-2 cross-infections among patients and oral healthcare workers [16].

In the initial phase of the pandemic, a 48.8% decrease in the number of pediatric dental patients treated was registered in Germany [17]. Similar findings have been reported in other countries [18,19,20,21,22,23,24,25] and attributed to the fact that dental settings and practices were generally perceived to be at high risk for SARS-CoV-2 transmission and thus unsafe, including by general dentists and pediatric dentists themselves [18,19,20]. Specifically, 60% of orthodontists felt that in-person monitoring for fixed orthodontic treatment should only be performed in emergencies, and in May 2020, 50% wanted to postpone aerosol-generating orthodontic procedures [26]. Accordingly, oral hygiene sessions, regular dental checkups, and aerosol-generating procedures were frequently postponed [21].

Table 3 summarizes the demand for pediatric dentistry and patient and parent/caregiver attitudes toward pediatric oral and dental care during the further waves of the COVID-19 pandemic in different countries [20,21,22,23,24,25,26,27,28].

#### 3.1.3. Pediatric Dentistry Practice: Infection Control, Ergonomics, and Pedodontics Patient Management

Guidelines for pediatric oral and dental care during the COVID-19 pandemic have been published by several pediatric dental associations, including the American Academy of Pediatric Dentistry (AAPD) [27,29], the Royal College of Surgeons England [30], and the Australian Dental Association [31]. In their guidelines, they have agreed on the indications and types of pedodontic treatment similar to those for the adult population [4].

The oral and dental needs to be addressed, the procedures to be performed, and the pediatric cases to be treated were reevaluated in terms of the priority of the procedures, with elective treatments deferred whenever possible [32,33].

Therefore, in addition to the need to address emergency and non-emergency pediatric oral and dental needs, the identification and potential treatment of oral mucosal lesions, potentially associated with SARS-CoV-2 infection, or detected in pediatric patients with COVID-19 because they are related to disease pathogenesis, complications, or treatment [22], became a new challenge in pediatric dental practice. Indeed, pedodontists play an important role in the detection of many pathologies that affect children at a young age and could benefit from early intervention [34].

In addition, treatment planning had to take into account procedure-related risk assessment, the distinction between aerosol-generating and non-aerosol-generating procedures, and the precise scheduling of surgical and follow-up sessions [7,23,24].

Moreover, the general health status of the pediatric patient also had to be considered when prioritizing dental treatment. In fact, pedodontic treatment of medically compromised patients with special needs could not be postponed. Untreated and unresolved oral and dental infections can have systemic recurrences, and oral and dental pain can negatively impact the overall health of the pediatric patient and the quality of life of patients and families, and even lead to self-injury. To maximize safety in this category of pediatric patients, especially those who have diabetes, are at risk for infective endocarditis, or are immunocompromised, the surgical session should be scheduled as the first appointment of the day in consultation with the patient’s family, preferably in a separate operating room, and disinfection and sanitization procedures should be performed with even greater care [4,29].

Furthermore, the AAPD recommended immediate care for pediatric patients with high caries index and dental emergencies according to specific guidelines.

In light of these considerations, in order to minimize the risk of infection in the pedodontic practice, on the one hand, and to optimize the management of pedodontic patients on the other, the pediatric dentist needed to streamline preventive and therapeutic strategies both in the office and at home [1,3,7,21].

##### In-Office Procedures

All known measures to control airborne infections, including SARS-CoV-2, have been incorporated into dental and pedodontic practice to ensure patient safety and allow pedodontic treatments to be performed while minimizing the risk of infection in dental settings [35,36,37,38].

Considering the frequent asymptomatic and oligosymptomatic course of COVID-19 in pediatric subjects and the virus incubation period in adult patients, all pedodontic patients had to be considered potential carriers of the disease [33,39].

Although essential, protective equipment that obscures facial expressions, such as visors and FFP-2 masks, limited verbal and nonverbal communication with pedodontic patients, which negatively affects behavioral management important for pediatric patients [21]. Considering that social isolation and disruption of routine activities can have physical and psychological consequences, including and perhaps, especially in children, and that behavioral management is even more important in pedodontic patients in the context of anxiety, fear, and pain, more attention should be paid in the COVID-19 era to gaining the trust and cooperation of young patients, both to humanize treatment and to reduce aerosol spread, which is generally greater in crying and agitated individuals [4,25]. Therefore, it may be helpful to prepare pediatric dentistry patients propaedeutically to see the wide variety of personal protective equipment provided by the surgeon and chairside assistant, which is then explained to him or her in person before the surgical session by means of a preventive long-distance conversation with the parents [26]. However, when behavioral management was inadequate, sedation and general anesthesia were feasible under specific indications in the COVID-19 era, which were explicitly rejected by the England Royal College of Surgeons in 2020 [4].

Although evidence is weak, irrigation before surgery with 0.2% povidone-iodine or hydrogen peroxide (1.5%) has been recommended [6,27,28].

To minimize aerosol generation during pedodontic procedures and thus the risk of SARS-CoV-2 transmission, nonrestorative treatments and minimally invasive aerosol-generating restorative techniques aimed at so-called biological caries management have been preferred [4]. Specifically, fluoride varnish, resin infiltration, and sealing have been recommended to treat asymptomatic, noncarious lesions in the primary and permanent dentition to halt their progression. For cavitated carious lesions in the primary and permanent dentition, alternative restorative techniques, temporary therapeutic restorations, silver fluoride, diamines, indirect pulp capping, and the Hall technique were recommended instead [4,29]. Ozone therapy has also been suggested as an alternative to aerosol-generating procedures for asymptomatic carious lesions [29].

Human and economic resources, as well as work agendas, have been ergonomically redesigned. The entire approach of oral, dental, and periodontal health of the pedodontic patient and pediatric oral and dental care has been redesigned from both therapeutic and preventive perspectives [7,30].

During the treatments, the number of in-person operative sessions was generally reduced and their duration was shortened whenever possible. Indeed, the AAPD encouraged maximizing treatment per operative session to reduce the number of visits [4].

Therefore, it seemed appropriate to favor more comprehensive and definitive approaches, opting for simpler and more predictable techniques and methods, associated with a lower risk of failure, and for less complex and articulated treatment plans, less exposed to the risk of complications and requiring multiple checkups [7].

Specifically, symptomatic caries of the deciduous and permanent dentitions were recommended to be medically approached, in the first instance [6]. In cases of coronal pulp involvement, agents inducing reparative dentin formation could be employed. Pulpectomy was indicated, instead, in case of root pulp involvement and preferably performed in a single rather than multiple operative session, thus efficiently reducing pediatric patients’ symptoms and exposure [6].

Oral and dental infections in pediatric patients can be at least initially treated with amoxicillin, phenoxymethylpenicillin, or metronidazole, administered up to three times daily unless otherwise indicated. Dental pain could be managed with paracetamol tablets (500 mg) or suspension (120 mg/5 mL or 250 mg/5 mL) given up to four times daily in age-dependent doses. The combination of paracetamol and ibuprofen could be prescribed only on the advice of the physician [6].

Nevertheless, in cases of severe pain and extensive destruction of dental tissue, tooth extraction was advocated to shorten the treatment time and reduce the risk of complications and subsequently repeated controls [31].

Considering the recommendation to shorten the contact time, it seemed appropriate to implement the communication with both pediatric patients and parents and the necessary support through teleconsultation, teleassistance, and telemonitoring, which are necessarily limited during the operative session [6].

On the contrary, the time between operative or face-to-face sessions was prolonged. In this perspective, the parents of the young patients, also with the help of distance education [1], assumed a central role in supporting pedodontic care in the home management of minor oral diseases and compliance with postoperative prescriptions and recommendations.

##### Home Care Prescriptions and Recommendations

Home care of pedodontic patients, which has always been considered essential for children’s oral health, became crucial during the first peak of the COVID-19 pandemic when regular dental care was suspended and remained so during subsequent pandemic waves of restricted and discontinued dental and pedodontic practice [17]. Indeed, introducing home oral and dental care in children with parental support could reduce the need for non-routine oral and dental check-ups and improve primary prevention strategies in general [1,7,32,33,34,35].

Specifically, the indication for the use of manual toothbrushes or electronic toothbrushes should be based on a case-specific assessment, as they are equally effective in controlling plaque accumulation [35,36]. Conversely, antimicrobial gels are a valuable addition to daily oral hygiene, as these products act on cariogenic and periodontopathogenic biofilm [37,38,39,40,41], reducing the risk of caries and gingivitis occurrence. In particular, gels containing chlorhexidine are more effective in controlling biofilm than gels containing fluoride [39,42]. However, the administration of chlorhexidine, regardless of the formulation (toothpaste, mouth rinse, gel), must be carefully evaluated after assessing the risk-benefit ratio for the individual pedodontic patient, as it is not free of side effects [37]. Alternatively, organic plant-based products can be recommended, which are also available in different formulas and have beneficial effects on plaque accumulation and gingival inflammation without causing adverse effects [38], although the available evidence is still controversial [39].

Probiotics, whose potential efficacy on cariogenic and periodontal pathogenic biofilms has been studied for several years [40], could also be promising adjuvants in long-term therapies such as orthodontic treatment. However, at the current state of knowledge, the type, dosage, and route of administration of probiotics, as well as the duration of supplementation that is most effective and at the same time safe for adult and pediatric patients, are still under investigation.

Oral hygiene instruction and motivational reinforcement of oral and dental care should and could continue to be delivered primarily via teledentistry tools and platforms, as described later.

### 3.2. Dental Caries in Pediatric Subjects during the COVID-19 Pandemic

Dental caries affects many adults and children worldwide. The prevalence of caries in primary dentition is estimated to range from 60 to 90% worldwide [41]. The variation in caries prevalence in pediatric subjects during the first and subsequent waves of the COVID-19 pandemic is difficult to determine. It may be underestimated due to reduced dental check-ups and the typical slow subclinical disease progression [43]. However, the incidence and progression of caries may have been negatively influenced by limited access to prophylactic treatments such as sealants and restorative therapies [44]. In this context, an increase in caries in deciduous teeth has also been pointed out [44].

In addition, dental caries is a multifactorial disease etiologically related to modifiable risk factors, among which the most important are lifestyles, which may have been influenced by the COVID-19 pandemic, negatively affecting the incidence of caries [45,46,47].

Lifestyle changes in adults and children who had to stay at home for an extended period of time have been investigated in several studies with discouraging results [17,44,46]. In general, sedentary lifestyles inevitably increased during home confinement, with 53.6% of the 220 pediatric subjects surveyed in the Docimo et al. study [44], reporting a reduction in daily activities, compared with 3.6% before the restrictions were imposed. In addition, time spent in front of the television increased by 3.66 ± 4.00 compared to 1.98 ± 1.13 h daily before the lockdown [47]. Sleep duration also changed, with 33.6% of children sleeping > 9 h per day compared to 14.1% before lockdown and a slight overall improvement in sleep quality (89.4% vs. 86.0% of children) who reported good sleep quality before and during the lockdown, respectively [46,47].

However, the changes in children’s lifestyles that accompanied the daily living restrictions and the suspension and subsequent restriction of pediatric dentistry may have indirectly led to an adaptive change in caries prevention strategies, generally guided by diet, oral hygiene, fluoride prophylaxis, regular dental examinations, and sealants, and thus may have affected the associated outcomes.

#### 3.2.1. Nutrition

Massive and frequent sugar consumption is one of the main risk factors for developing dental caries, as bacteria such as *Streptococcus mutans* and *Lactobacillus casei* can produce fermented acids that lead to the dissolution of enamel from sugars [48]. In fact, the World Health Organization (WHO, Geneva, Switzerland) has strongly recommended reducing the consumption of free sugars throughout life. It should represent less than 10% of total energy intake, preferably less than 5% in children and adults [49]. Notably, non-sweet snacks such as French fries, French potato fries, popcorn, and other low-nutrient, high-calorie foods are also potentially cariogenic [50,51].

Nonetheless, unhealthy changes in children’s eating habits occurred frequently during the COVID-19 pandemic due to new daily schedules, distant school hours, parents working at home, and the economic instability that affected many families [17,45]. In addition, pediatric subjects forced to stay home by current regulations were also limited in their ability to participate in sports, which often led to negative body weight regressions [45]. In addition to these factors, the COVID-19 pandemic undoubtedly had psychological effects on children that contributed to changes in quantity, quality, and/or frequency of food intake [46,47].

In this context, two opposite trends have been noted in the literature [46]: on the one hand, children’s diet seems to improve during the closure, as parents have more opportunities to cook healthy foods [17], and on the other hand, the frequency of food intake and the consumption of carbohydrates and snacks generally increases [49]. The choice of foods purchased, with spending on fresh foods such as fruits, vegetables, and fish decreasing in favor of canned foods, was likely influenced by restrictions on daily shopping during the COVID-19 curfew [48].

In addition, eating outside meals and consuming sweets increased from 25% before the lockdown to 54% during the lockdown, although 50.9% of participants reported that they had not negatively changed their eating habits [46,48]. Accordingly, Turkish children’s diets improved during quarantine relative to parental attitudes [17], increasing the frequency of eating fresh fruit and vegetables, paying attention to cariogenic foods, avoiding fast food and prepackaged food, and reducing carbohydrates [17]. In contrast, of the 1003 parents of children aged 0–12 years surveyed in the study by Campagnaro et al., 61.5% reported that their children’s eating habits had changed during the pandemic, mainly due to an increase in the amount of food [51].

In addition, the indirect effects of parents’ attitudes toward food quality, quantity, and frequency on the dietary style of pediatric subjects should also be considered [52]. Adverse changes in dietary habits during the COVID-19 pandemic were also associated with the low education level and the male gender in a Chinese sample [45].

#### 3.2.2. Oral Hygiene

Children’s oral hygiene routines did not appear to have changed during the lockdown, and 75.0% of children continued to brush their teeth, mainly before bedtime [52].

In 15.0% of cases, the frequency of oral hygiene was reduced, and 10% did not perform it every day [44]. Such results in the youngest subjects could be due to parents spending more time at home. Accordingly, mothers of 328 children reported that 71.7% of their children brushed their teeth adequately during the lockdown, and parental supervision of children’s oral hygiene increased to 61.3%, compared with an estimated 30.2% before the pandemic [17].

In contrast, a slight deterioration in oral hygiene practices was noted among adolescents, with 39.1% brushing their teeth more than three times daily during the lockdown, compared with 47.8% before the lockdown; notably, 52.2% reported brushing their teeth less than three times daily before the lockdown, compared with 60.9% during the lockdown [47].

#### 3.2.3. Fluoroprophylaxis

Topical and systemic fluoride prophylaxis is one of the most important strategies to prevent caries development, and there is sufficient evidence that the use of fluoride is the best means of caries prevention in school-aged children [42].

Fluoride toothpaste is one of the most widely used forms of topical fluoride prophylaxis at home and the easiest to implement during the COVID-19 pandemic when government restrictions hampered professional fluoride prophylaxis. However, compared with pre-pandemic estimates, no differences were found in the frequency of fluoride toothpaste use, which was reported as “often” by 55.0% and “always” by 33.6% of participating pediatric subjects [44].

### 3.3. TMD Disorders and Oral Bad Habits

During the lockdown, TMDs worsened uncontrollably in 60% of cases, and more than 20% of dentists reported being contacted by telephone by patients for temporomandibular problems [18]. Furthermore, at the end of the first lockdown in May 2020, more than 1500 Italian TMD patients were found to have a general worsening of anxiety, stress, and depression, which was associated with preauricular pain or temporomandibular jaw pain in 20% of cases [52]. This prevalence of temporomandibular pain and noise was significantly higher compared to pre-pandemic estimates (16%) [22] and even worsened to 45% in a comparable sample one year later, in March 2021 [27]. Pediatric dentists have also reported a similar increase in Germany in the context of tightened social restrictions to combat SARS-CoV-2 infection [21].

Few studies have instead examined the changes in poor oral habits associated with the pandemic scenario COVID-19 to consider them as reflecting what has been termed “repetitive physical behavior,” mainly due to stress and anxiety associated with recent lockdown and quarantine periods. According to the sparse literature, the results varied by age. While adults showed an increase in nail-biting, lip-sucking, and finger-sucking after the peak of COVID-19 and corresponding curfews [52,53], children reported that the same oral bad habits before curfew significantly decreased after curfew, presumably due to the close and sustained “reassuring” presence of parents [54,55].

### 3.4. Pediatric Oral and Dental Care: Lessons Learned from COVID-19 and Future Applications in Pedodontics

The strategies initially developed to prevent pedodontic patients from being involuntarily abandoned and to ensure safe pediatric oral and dental care, especially during the first waves of the COVID-19 pandemic, consisted primarily of working with patients’ parents to ensure their compliance with postoperative prescriptions and general oral health recommendations.

In particular, parents’ home-based treatment of minor oral diseases and long-term monitoring of treatment by pediatric dentists [56] were innovatively supported by making teledentistry the focus of secondary prevention efforts.

Parent education and pediatric patient motivation enhancement, which target oral health literacy, motivation, and risk factor control as part of primary prevention, also benefited from e-medicine applications [57].

Thus, along with improved infection control, teledentistry tools and applications have played a key role in supporting pediatric oral and dental care [1] in secondary and primary prevention in the COVID-19 era.

This trend may continue in the post-COVID-19 era by integrating applications of teledentistry into pedodontic practice [1], particularly in the care of medically vulnerable patients, within appropriately developed policies and guidelines related to data protection, legal issues, financial implications, and privacy [57,58].

#### 3.4.1. Pediatric Dentistry Practice during and after the COVID-19 Era

Considering the frequent asymptomatic and oligosymptomatic course of COVID-19 in pediatric patients and the incubation period of the virus as in adult patients, all unvaccinated pediatric subjects may be even more exposed to infection.

Therefore, preventive measures to control airborne and aerosol-transmitted infections in dental settings should be continuously incorporated into pedodontic practice, at least for the time being, in addition to standard measures, to ensure the safety of unvaccinated pediatric patients [37,38] and to avoid the so-called “pandemic of the unvaccinated” [59].

Accordingly, appropriate pre-check triage should continue to be performed [60], and rapid diagnostic testing for SARS-CoV-2 antigens and antibodies may be proposed in the near future before the treatment of all patients [59].

The measures described above, which could be supported in the future by the widespread use of newly developed biotechnological control measures [33] should be explicitly maintained in the event of a sporadic recurrence of infection in regional nests or areas where the pandemic may last for months.

On the other hand, several recurrent epidemics and pandemics have already changed dental practice. Further epidemics and pandemics can be expected to arise unpredictably from complex, changing ecosystems that are often far from control or regulatory surveillance, such as the current outbreak of human Monkeypox [61].

Given the fortunately low but possible occurrence of oral lesions after anti-SARS-CoV-2 vaccination, pediatric dentists should be alert to such possible additional oral needs [22,62].

Consistent with WHO, prevention and oral self-care remain a high priority during the COVID-19 pandemic [49].

#### 3.4.2. Teledentistry

Teledentistry is a field of telemedicine in which technological devices are used for dental care [55,56,63]. It is defined as “the remote practice of dentistry by dental professionals within the confines of their practice through the use of information and communication technology” [64]. Remote dentistry was widely practiced before the COVID-19 pandemic, and the primary purpose of remote consultations was originally to improve health care in rural areas, increase access to dental care, provide rapid consultations, and reduce inappropriate referrals, thereby reducing the cost of dental care, shortening waiting lists for checkups and consultations, and eliminating social and geographic disparities [55,56,65].

During the COVID-19 pandemic, maintaining good oral health in children became particularly important given the circumstances that limited the provision of regular dental services [1]. In these circumstances, teledentistry enabled the provision of health services remotely via mobile phones and wireless technologies [66]. The widespread use of teledentistry during the COVID-19 pandemic aimed to limit the chain of infection by avoiding cross-infection [1], while continuing to counsel, diagnose, treat, and educate patients about oral health [66], despite the inherent limitations of the service [67].

Subsequently, interest in teleservice continued to grow, and various applications were explored, including storing data and sharing it with dental practitioners [68,69], assisting and monitoring patients remotely, including through live video sessions between dentist and patient, and providing virtual video technology for oral health [69] or mobile devices.

**Figure 1:**
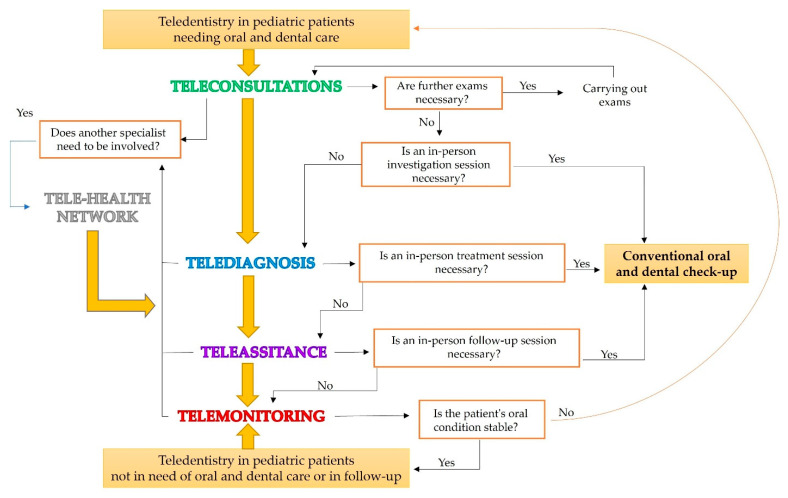
Teledentistry workflow and related applications in pediatric oral and dental care.

Future applications in pedodontic practice have begun to emerge. These include teleconsultation, telediagnosis, telescreening, and telehealth networks that will be permanently integrated into pediatric oral and dental health management, as shown in Figure 1.

##### Teleconsultations in Pediatric Dentistry

Teleconsultations were the most popular form of Teledentistry service delivery by parents, caregivers, or school teachers for children [8]. The study by Sanghvi et al. [70] showed that 74% of respondents preferred a teleconsultation via video call over a traditional phone call. The virtual face-to-face relationship actually simulates a regular dentist-patient interaction [70]. It allows the patient to show via video oral cavity problems and concerns that would otherwise only be described by the patient himself.

In addition, teleconsultations conducted via live video have been shown to provide good predictability in assessing the best intervention for children with obvious dental disease [71]. Indeed, planned pediatric dental interventions were successfully delivered in 88% of children initially visited with a video consultation [71].

Thus, teleconsultations can be successfully used for treatment decision making by determining the treatment to be performed with good predictability. Consequently, teleconsultations can reduce the number of traditional dental visits in adverse situations, such as during the COVID-19 pandemic. Accordingly, Wallace et al. [59] pointed out how telephone consultations were reduced by more than a third of in-person appointments between 13 May and 12 June 2020.

##### Telediagnosis in Pediatric Dentistry

In telediagnosis, the electronically transmitted information, along with photographic and radiographic documentation and laboratory tests, is sent by the responsible caregivers to the pediatric dentist [8].

In the study by Sanghvi et al. [70] it was reported that 100% of the dental patients surveyed preferred a telephone visit during the COVID-19 pandemic. In comparison, only 32% of the respondents also preferred this service before the pandemic.

In addition, the study by Haron et al. [72] examined the sensitivity and specificity of telediagnoses performed by specialists when oral mucosal lesions were documented via mobile phone cameras. A sensitivity > of 80%, a specificity of 87% in categorizing the type of oral lesion, and an agreement of 85% when the same lesion had been assessed by clinical oral examination were found [72].

However, telediagnosis can be helpful in medical data collection, especially in medically compromised pediatric patients. It can provide initial clinical information about oral and dental problems to prepare children and parents for the in-person examination. This is especially beneficial in cases of dental trauma, caries, and malocclusion.

##### Telescreening in Pediatric Dentistry

Telescreening for dental caries, which has been studied in high-risk individuals with cleft lip and palate, helped provide immediate support and professional reassurance and thus has been suggested as a valuable method for prevention and follow-up [73].

Dental caries is a preventable disease that can lead to significant morbidity, which in turn requires costly treatment if left untreated [74,75]. Therefore, regular oral and dental screenings and health education have great potential to improve oral health and save significant resources. Because telediagnostics have comparable diagnostic performance to conventional methods, they can be permanently integrated into the standard screening process, going beyond a practical and economically viable population-based approach in remote or rural and underserved urban communities [65,76], particularly in medically compromised pedodontic patients and those with psychomotor disabilities.

##### Teleassistance and Telemonitoring in Pediatric Dentistry

Teleassistance and telemonitoring in orthodontics during the COVID-19 pandemic effectively managed minor emergencies and provided regular check-ups [77]. Therefore, both means should be considered to achieve an acceptable compromise for pedodontic service delivery during high infection rates [70].

Moreover, teleassistance and telemonitoring could be successfully employed under further research even following the pandemic. Indeed, telemonitoring may allow routine checkups to be limited by remote visits, and can be used both to prevent or monitor preexisting pathological conditions and to monitor treatment response and progress Telemonitoring in orthodontic patients has also been evaluated by Sangalli et al. [78], who highlighted the improvement in biofilm control and consequently caries prevention.

Furthermore, telemonitoring may indirectly help to reduce the number of check-ups and inappropriate referrals, thus shortening waiting lists [31,77,79].

##### Telehealth Networks in Pediatric Dentistry

Telehealth networks that link physicians and dentists from different specialties to discuss diagnoses and complex multidisciplinary treatment plans may solve more clinical cases [55,67].

In addition, sharing patients’ clinical, photographic, and radiographic records and data could allow better integration of subsequent therapeutic phases in multidisciplinary treatments and better align surgical sessions. Such an integrated approach could be particularly beneficial in complex cases.

##### Tele-Education and Pediatric Dentistry

Patient education about home care through teleassistance and home instructional videos has been studied more than once in pediatric patients undergoing fixed orthodontic treatment [80] and showed a successful reduction of plaque accumulation. These results, which may be because patients were also able to view the educational videos more than once at home, showed better patient cooperation with oral hygiene and thus may also indicate higher overall treatment compliance.

Online training offers more and more efficient educational opportunities for oral healthcare providers [81,82] and dental students [60] and reduces, at least in part, the time and cost involved.

Future activities for targeted education and training on teledentistry approaches, applications, and mobile health tools should be considered [83].

#### 3.4.3. Mobile Health (m-Health)

Due to the increasing popularity of mobile devices, their low cost, and the greater possibility of Internet connectivity, several medical applications, known as m-health applications [2], also concerning dentistry, have also proliferated in recent years, which can also be downloaded to patients’ smartphones and have proven particularly useful in pandemic times.

Digital technologies have become integral to daily life, and the population is highly connected worldwide. Consequently, the pace of innovation in teledentistry is accelerating the development of mobile health (m-health) technologies that benefit patients and the healthcare system.

**Figure 2:**
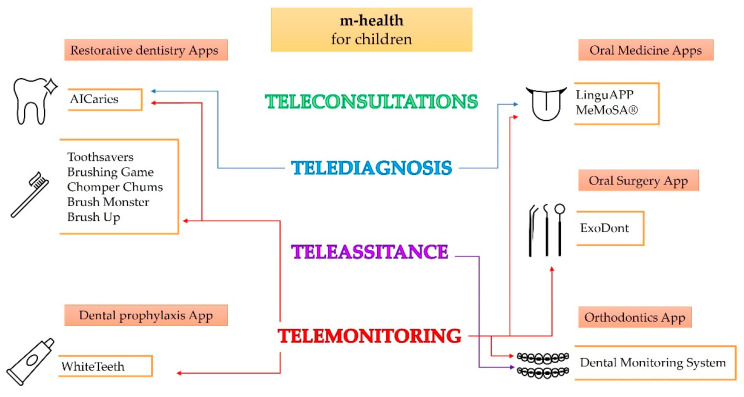
M-health solutions and related applications in pediatric oral and dental care.

Accordingly, more and more research is being conducted on the attitudes and expectations of dentists and pediatric dentists regarding m-health and teledentistry in general to determine the proper selection and use of the digital mHealth solutions already available and to develop more [55,63], to integrate m-health technologies into the routine of pediatric dentistry (Figure 2).

##### Restorative Dentistry Apps

Considering the unfavorable circumstances caused by an increase in the number of SARS-CoV-2 positive cases, oral health prevention in children also uses remote communication tools to educate children, parents, caregivers, schools, and health workers who come into contact with children on a daily basis on how to maintain good oral health [1]. During the COVID-19 pandemic, tools from IT were among those used to promote good oral hygiene practices, particularly among the pediatric population [66].

Several cell phone apps have been developed to promote oral health prevention, including many oral hygiene game apps (e.g., Toothsavers Brushing Game, Chomper Chums, Brush Monster, Brush Up) for young patients to play with [84]. The purpose of these apps is to playfully simulate the behaviors and techniques for good oral hygiene to educate the child, who could then transfer the game to their oral health [84].

To monitor the potential development of new carious lesions in children, an artificial intelligence-driven smartphone app called AICaries was developed [85]. The AICaries app uses an algorithm-based tool that detects carious lesions, while other components assess the caries risk of the parent and child. With AICaries, parents are also informed about the risk factors associated with caries so that they can reduce the same risk factors in their children [85].

##### Dental Prophylaxis App

The WhiteTeeth Mobile App was developed to promote good oral hygiene in young patients undergoing orthodontic treatment [86], Patients’ information about their oral hygiene behavior and motivation to follow good hygiene practices is collected during registration.

The app prompts the patient to use the reveal tablets, which stain plaque red, and then take a selfie using the same app [86]. When the patient clicks on the colored areas of their selfie, they learn how much plaque has accumulated in that area [86]. The recording of oral health behavior is queried at different times, so the number of clicks recorded acts as positive reinforcement. In addition, the app provides further positive reinforcement in the form of short videos based on documented information [86]. In patients who used the WhiteTeeth app in addition to their usual home care, a randomized controlled trial observed a significant decrease in gingival bleeding, plaque accumulation, and the number of plaque-covered sites, as well as a concomitant increase in oral fluoride use, compared with subjects who did not use the app [86].

Given these promising results, the WhiteTeeth app could serve as a positive reinforcement of oral hygiene and therefore be suggested as part of home care recommendations for all pediatric patients, especially during the pandemic season when regular dental checkups and prophylaxis sessions could be interrupted and stretched out in time.

##### Oral Medicine Apps

LinguAPP is an app that can be downloaded on Android and iOS devices and requires patients to attach at least two photos and, if desired, videos of the oral manifestations for which they need advice [2]. The patient is also asked to complete a triage questionnaire, which provides additional important information for the physician to understand the patient’s clinical condition [2]. The system sends an email to the specialist, who can make a preliminary diagnosis via the Internet. With LinguAPP, making a preliminary diagnosis, discussing treatment options, and giving the patient follow-up appointments are possible. If deemed necessary, it is also possible to ask the patient for an in-person appointment [2].

Another phone app mainly used for the early detection of oral cancer is the Mobile Mouth Screening Anywhere (MeMoSA**^®^**) app, which allows oral lesions to be documented via the app using images taken with a phone camera and provides additional information in the form of text [72]. In addition, the dentist can manually add demographic information, risk factors, and signs suggestive of oral cancer [72,86]. Even though the risk of developing oral cancer is higher after the second half of the fourth decade of life [87], it should not be underestimated in children. Indeed, unfortunately, an increasing number of reported cases diagnosed with oral cancer among pediatric subjects under 16 years of age (8 cases between 1970–1999 vs. 17 cases between 2000–2020) has been described [88]. MeMoSA**^®^** could therefore be considered as an additional tool for families to monitor the oral health of pediatric patients when routine visits are limited or completely interrupted.

##### Oral Surgery App

ExoDont is an m-health application that can be downloaded on Android devices to assist patients in the postoperative period after a surgical procedure [89]. It serves as a memory aid, reminding patients of postoperative instructions and when to take their medications according to the timing and dosing instructions provided by the physician [89].

The ExoDont app aims to improve patient adherence to postoperative instructions and pharmacological treatments [89], which critically impacts recovery and the risk of complications [90,91]. In this scenario, ExoDont could be a valuable tool for children and adults. The reduction in postoperative complications would allow the specialist to monitor the patient remotely and minimize both operative and follow-up visits.

##### Orthodontics App

Dental Monitoring System is a newly introduced m-health application that enables monitoring the progress of orthodontic treatments with clear aligners by combining a dental monitoring system (DM) with artificial intelligence [92].

The patient can use the Dental Monitoring System to perform the monitoring required for each aligner change remotely. For this purpose, the patient is provided with a retractor and a DM ScanBox© that guarantee good quality intraoral photos via the personal smartphone. With this system, the dentist can assess the function and fit of the aligners as well as the status of the attachments, buttons, and elastics [92]. In addition, the system DM provides follow-up information to the patient based on the treatment plan. The patient receives the “GO” signal when ready to move to the next aligner rather than the “NO GO” signal in the opposite case. In the latter case, the patient must perform new scans in the following days until he/she receives the signal “GO” [92].

After the patient performs the oral scan, the acquired data are transmitted via a remote server to the orthodontist, who can decide whether a face-to-face session is required or the treatment can be continued remotely [92]. In this way, the orthodontist can always decide whether to call the patient back for a visit, even if he receives a “GO” signal from the system.

The Dental Monitoring System thus makes it possible to minimize the frequent visits of the pediatric patient treated with clear aligners, thus reducing the possibility of cross-infections for the child, the dentists, and their families.

## 4. Discussion

In addition to the direct impact of SARS-CoV-2 infection, the COVID-19 pandemic had numerous effects on human health and psychophysical well-being [21,33]. Regarding oral health, during the curfews and quarantines of the COVID-19 pandemic, routine dental care was suspended or postponed [1], and dental procedures were mainly limited to dental emergencies [2]. Therefore, the absence of routine checkups, changes in dental hygiene [51] and dietary habits [22], and fear of transmission of SARS-CoV-2 in dental offices [22] were responsible for the medium- and long-term direct and indirect effects of COVID-19 on people. In addition, children were disrupted in their social and daily activities, which contributed to increased anxiety and stress [21].

In the present work, the changes in pediatric oral and dental care during and after the outbreak of COVID-19 were analyzed from a pedodontic perspective.

During the initial phase of the pandemic, the demand for pediatric dental care decreased significantly for all scheduled medical appointments and preventive checkups [16]. The availability of treatment in private practices and hospitals was significantly reduced [16] as all medical resources were used to combat the then-unknown infection. In addition, during the curfew, emergency and urgent dental care were the prominent cases admitted to hospitals and facilities, which experienced an increase in admissions for early childhood caries, dental pain, facial swelling, and dental trauma worldwide, largely due to the unavailability of private and outpatient structures [8,9,10,11,12]. The restriction of dental care was justified to contain the spread of SARS-CoV-2 and to limit the consumption of personal protective equipment, which was initially in short supply. From an ethical perspective, limiting dental care was an ethical compromise for a greater good [93]. However, the closure of all dental services, except for emergency services, during the first 4–6 weeks of the pandemic [94] raises the ethical doubt that institutions did not attach importance to dental care and the maintenance of oral health and thus the general health of the population [95].

The ethical doubts are reinforced by the fact that the decision to limit dental care had had negative consequences, especially in certain geographic areas or socioeconomic contexts where, even in times when dental care was free for children, negligence on the part of parents and children in performing dental examinations was observed [14,95]. Consequently, in an environment already characterized by unpredictable socioeconomic factors, the limitation of dental and pedodontic care during the COVID-19 pandemic further contributed to the deterioration of oral health in children [12].

Later, after SARS-CoV-2 was identified and the mechanisms of infection were known [96], several measures to control airborne infections and operational policies and procedures to avoid aerosol-generating practices were introduced [70]. However, given the frequent asymptomatic and oligosymptomatic course of COVID-19 in pediatric patients, the variable incubation period, and the rapid emergence of SARS-CoV-2 variants, the number of infections in the pediatric population increased substantially in parallel with the removal of restrictive measures, despite the availability of vaccination [32,33]. Fortunately, the number of deaths and severe forms of COVID-19 in children remained low [17,18].

All these circumstances have led to the dental office being considered a risky place [22], contributing to delayed treatments and worsening oral conditions. In this scenario, preventive dentistry has failed, and tertiary and secondary prevention has increased.

In addition, interrupted long-term treatments, unfinished therapies, or newly developed pathological conditions have had to be managed, and patient and parent concerns have had to be addressed to ensure the continuity, timeliness, and, consequently the effectiveness of pediatric oral and dental care.

In parallel, global conditions have imposed on physicians and dentists the need to develop new communication skills based on telemedicine in general and teledentistry in particular [1,2,9,72]. Dentistry has not only reorganized itself in priorities and ergonomics but has also had to develop new workflows to maintain contact with its patients, avoiding risks and widespread infections on the one hand and diagnostic and therapeutic delays on the other. In this context, teledentistry has evolved into a series of e-medicine applications and digital dentistry for the remote treatment of patients [2,66,84,85,86,90,91,92], with a reorganized and redesigned way of working to support pediatric oral and dental care in secondary and primary prevention. Future applications in pedodontic practice have gradually emerged, including teleconsultations, telediagnosis, telescreening, and telehealth networks to be permanently integrated into pediatric oral and dental health management.

## 5. Conclusions

The COVID-19 pandemic has irrevocably impacted our daily lives in many ways and has set points from which there is no turning back, only forward.

Based on the good that this world disaster has brought about, we can look at the technological and operational achievements as a starting point to maintain and develop a “2.0 approach” to oral health that increasingly perfects and focuses on remote dentistry so that remote rural or inaccessible areas can be reached instead of limiting human contact to meet each patient without feeling abandoned, isolated or unsafe.

## Figures and Tables

**Table 1:** Emergency and urgent oral and dental needs requiring pedodontic care during the first peak of the COVID-19 pandemic.

	US [9]		Italy [10]		Turkey [11]		Israel [12]	
	2019	2020	2019	2020	2019	2020	2019	2020
Dental traumas	67.4%	68.0%				Dislocation and Avulsion 41% Fractures 35.5%		24.7%
School-related	21.8%	1.9%						
Cycling-related	3.6%	9.6%						
Dog bites-related	6.0%	8.7%						
Swelling/pain								32.6%
Swelling due to abscess	22.7%	25.2%				86.5%		
Pain due to dental pulp inflammation						94.0%		
Infections	28.1%	23.5%	15%	15%				28.4%
Dental	40.0%	68.1%						
Mucosal (“oral ulcers”)	47.6%	19.4%						
Mucosal injuries								
Due to orthodontic appliances								20.5%
Not due to orthodontic appliances (any)								19.5%

**Table 2:** Oral and dental treatments performed on pediatric patients during the first peak of the COVID-19 pandemic.

	India March–July 2020 [14]	Poland March–April 2019/2020 [15]	
**Non Emergency treatments**		2019	2020
		Primary teeth	
		6.4%	19.3%
Teeth restorations	42%	Permanent teeth	
		5.8%	11.4%
Preventive procedures	24.4%		
Elective treatments	12.6%		
**Emergency treatments**			
Endodontics		3.2%	12.8%
Abscess incisions		3.5%	17.8%
Oral mucosal lesions treatments		2.3%	4.3%
Surgical dressing		1.5%	10.07%

**Table 3:** The demand for pediatric dentistry and patient and parent/caregiver attitudes toward pediatric oral and dental care during the further waves of the COVID-19 pandemic.

	Turkey [20]		Germany [21]	Brazil [22]		Italy [23] (Orthodontics)		Italy [24]
Dental routine visits	Before COVID-19 outbreak 60.4%	After COVID-19 Outbreak 5.2%		Before COVID-19 outbreak 24.4%	After COVID-19 outbreak 17.8%			
Missed dental routine visits		64.2%				16%		
Dental treatment			−38%					
Urgent treatments (only)					66.6%			72%
Non-urgent treatments					15.1%			
Missed appointment for urgent treatments					86%			
Parents’ anxiety or fear	54.6%			65.3%				
Parents considering the dental setting as a source of infection	79.6%					2020 [24] 55.3%	2022 [25] 43%	
